# The changes of the interspace angle after anterior correction and instrumentation in adolescent idiopathic scoliosis patients

**DOI:** 10.1186/1749-799X-2-17

**Published:** 2007-10-29

**Authors:** Yipeng Wang, Guixing Qiu, Bin Yu, Jianguo Zhang, Jiayi Li, Xisheng Weng, Jianxiong Shen, Qi Fei, Qiyi Li

**Affiliations:** 1 Department of Orthopedics, Peking Union Medical College Hospital, Chinese Academy of Medical Sciences & Peking Union Medical College, Beijing, China

## Abstract

**Background:**

In idiopathic scoliosis patients, after anterior spinal fusion and instrumentation, the discs (interspace angle) between the lowest instrumented vertebra (LIV) and the next caudal vertebra became more wedged. We reviewed these patients and analyzed the changes of the angle.

**Methods:**

By reviewing the medical records and roentgenograms of adolescent idiopathic scoliosis patients underwent anterior spinal fusion and instrumentation, Cobb angle of the curve, correction rate, coronal balance, LIV rotation, interspace angle were measured and analyzed.

**Results:**

There were total 30 patients included. The mean coronal Cobb angle of the main curve (thoracolumbar/lumbar curve) before and after surgery were 48.9° and 11.7°, respectively, with an average correction rate of 76.1%. The average rotation of LIV before surgery was 2.1 degree, and was improved to 1.2 degree after surgery. The interspace angle before surgery, on convex side-bending films, after surgery, at final follow up were 3.2°, -2.3°, 1.8° and 4.9°, respectively. The difference between the interspace angle after surgery and that preoperatively was not significant (P = 0.261), while the interspace angle at final follow-up became larger than that after surgery, and the difference was significant(P = 0.012). The interspace angle after surgery was correlated with that on convex side-bending films (r = 0.418, P = 0.022), and the interspace angle at final follow-up was correlated with that after surgery (r = 0.625, P = 0.000). There was significant correlation between the loss of the interspace angle and the loss of coronal Cobb angle of the main curve during follow-up(r = 0.483, P = 0.007).

**Conclusion:**

The interspace angle could be improved after anterior correction and instrumentation surgery, but it became larger during follow-up. The loss of the interspace angle was correlated with the loss of coronal Cobb angle of the main curve during follow-up.

## Background

With improved recognition of adolescent idiopathic scoliosis (AIS), the treatment became more and more standardized. Different scoliosis needs different surgical approach. For thoracolumbar and lumbar scoliosis, anterior spinal fusion and instrumentation has been used for many years. After anterior spinal fusion and instrumentation, the disc between the lowest instrumented vertebra (LIV) and the next caudal vertebra became more wedged. The angle between the inferior endplate of the LIV and the superior endplate of the next caudal vertebra is called interspace angle[1,2]. Some doctors reported that this angle usually became larger during follow-up. We reviewed the results of the patients that underwent anterior spinal fusion and instrumentation between November, 1998 to May, 2003 in our hospital, and analyzed the changes of the interspace angle.

## Methods

We retrospectively reviewed the AIS patients that underwent anterior spinal fusion and instrumentation since November, 1998 to May, 2003 in our hospital. The inclusion criteria were as follows:(1) idiopathic scoliosis;(2) age not over than 18 years old;(3)thoracolumbar scoliosis or lumbar scoliosis(PUMC classification Ib/Ic; Lenke classification type 5), or thoracic scoliosis and lumbar scoliosis, but the thoracic curve was flexible and selective anterior spinal fusion and instrumentation of the lumbar curve could be performed (PUMC classification IIc1/IId1; Lenke classification type 5) [3,4];(4) single anterior approach;(5)at least 6 months follow-up.

Measuring the standing anteroposterior(AP) film, lateral film, supine Bending films of the full spine preoperatively and the standing AP and lateral films of the full spine post-operatively and at final follow-up, recorded the coronal Cobb angle, flexibility of the curves, correction rate, apical vertebral rotation(AVR) and apical vertebral translation(AVT), and coronal balance(CB). The interspace angle on preoperative AP film, Bending films, post-operative AP film, at final follow-up AP film were also recorded. If the angle was opened toward the convex side of the scoliosis, we assigned it as "+", otherwise "-". The vertebra rotation was according to Nash-Moe method[5], and the details were as followed:

0 rotation had no asymmetry of either the position or shape of either pedicle;

1+ had medial migration of the convex pedicle limited to the most convex segment selected, and there was slight flattening of the oval of both pedicles with the concave border of the concave pedicle starting to disappear;

2+ rotation had further migration of the convex pedicle into the second convex vertebral segment while the concave pedicle gradually became indistinct;

3+ rotation was obtained when the convex pedicle reached the mid-line and was completely contained by the third segment;

4+ rotation occurred as the convex pedicle passed through the mid-line into the fourth segment on the concave side of the body.

According to the definition of Scoliosis Research Society, AVT was defined as the perpendicular distance in millimeters from the midpoint of the apex to the plumb line drawn from the spinous process of C7 for the thoracic curve, or to the central sacral vertical line (CSVL) for the lumbar curve on standing AP films, and the coronal balance was defined as the horizontal distance of the midpoint of the C_7 _from CSVL on standing AP films[6].

We used SPSS 10.0 software for statistical analysis. T test was used. Pearson's correlation coefficient(r) was calculated to analyze the linear correlation. A value of *P *< 0.05 was considered statistically significant.

## Results

**Figure 1:**
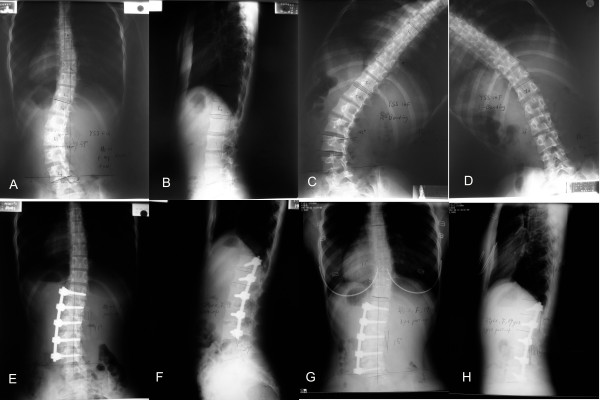
A 16-year-old female, AIS, PUMC IId1 type. Preoperative X-ray showed a 39° left lumbar curve and a 20° right thoracic curve and the apex of the lumbar curve was located at L_2,3 _disc. Preoperative lateral X-ray showed no thoracolumbar kyphosis (A, B). The interspace angle between L_4 _and L_5 _was 4°. On the right Bending film, the interspace angle was 9° (C). On the left Bending film, it turned to 0° (D). Anterior correction and fusion was performed, and the fusion level was from T_12 _to L4. Postoperative films showed a good correction and the interspace angle was improved to 0° (E, F). Three-year post-operative follow-up, the interspace angle increased to 2° with also a good coronal balance(G, H).

Thirty patients were included, 4 male, 26 female, with an average age of 14.8 years old (range, 10~18 years). The mean follow-up time was 17.7 months (range, 6~42 months). Single thoracolumbar or lumbar curve 8 cases, thoracic and lumbar curve 22 cases, which included PUMC classification type Ib 3 cases, Ic 5 cases, IIc1 2 cases, IId1 20 cases(Fig. 1). The main curves were toward left in 25 cases and right in 5 cases. We selected combined thoracic and abdominal approach or retroperitoneal approach to perform anterior correction and fusion surgery and standard derotation was performed. The selection of fusion level was according to Hall's principle, and the disc below and above the fusion level should be mobile[7]. The instrumentations included: Texas Scottish Rite Hospital instrument(TSRH) 9 cases, Cotrel-Dubousset Horizon(CDH) 12 cases, Moss-Miami 8 cases, Isola 1 case. The fusion levels were as follows: T_10_~L_2 _2 cases, T_11_~L_2 _1 case, T_10_~L_3 _2 cases, T_11_~L_3 _4 cases, T_12_~L_3 _15 cases, T_12_~L_4 _6 cases. The LIV were located at L_2 _in 3 patients, L_3 _in 21 patients and L_4 _in 6 patients. Thus the interspace angle were located at L_2,3 _in 3 patients, L_3,4 _in 21 patients and L_4,5 _in 6 patients.

The mean pre-operative coronal Cobb angle of the main curve was 48.9° (range, 33°~62°), on convex side-Bending film, it turned to 16.1°( range,-15°~40°), with an average flexibility of 67.2%(range, 31.0%~100%). In the patients with double curves, the mean coronal Cobb angle of the second curve was 31.3°( range, 20°~48°), on convex side-Bending film, it turned to 12.1°( range, 2°~24°), with an average flexibility of 62.0%(range, 16.7%~92.9%). After surgery, the mean coronal Cobb angle of the main curves was11.7°( range,-1°~36°), with an average correction rate of 76.1%(range, 40%~100%). In the patients with double curves, the mean coronal Cobb angle of the second curves was19.1°( range, 10°~32°) after surgery, with an average correction rate of 38.8%(range, 3.0%~60%). The coronal Cobb angle, AVR and AVT of the main curve were significantly improved after surgery. The details of the parameters of the main curves before and after surgery were list in table 1.

**Table 1:** Changes of the scoliosis parameters of the main curves before and after surgery(X ± S)

parameters	pre-operation	post-operation	t value	P value
Coronal Cobb angle	48.9° ± 9.2°	11.7° ± 8.8°	19.76	0.000
Coronal balance(mm)	14.1 ± 12.0	15.6 ± 13.0	0.66	0.516
AVR(degree)	2.1 ± 0.6	1.2 ± 0.5	9.36	0.000
AVT(mm)	43.3 ± 13.2	13.4 ± 9.0	10.63	0.000

**Figure 2:**
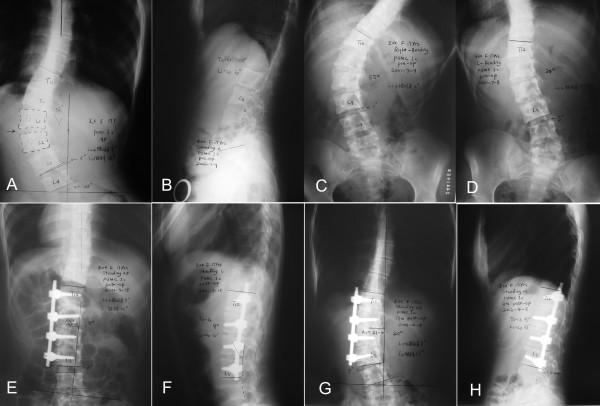
A 17-year-old female, PUMC Ic type. Preoperative X-ray showed a 52° left lumbar curve with the apex at L_1,2 _disc(A, B). The interspace angle between L_3 _and L_4 _was -5°. On the right Bending film, the interspace angle was 0°(C). On the left Bending film, it was still -5°(D), and T_12_-L_1_and L_2,3 _disc didn't open, so the fusion should include T_12 _to L_3_. Postoperative films showed a good correction and the interspace angle improved to 0° (E, F). Nine months later, there were some loss of the correction and the interspace angle increased to 7° (G, H).

The changes of the interspace angle before and after surgery were list in table 2. The mean interspace angle was 3.2° preoperatively, after surgery, it was corrected to 1.8°, but the difference was not significant (t = 1.146, P = 0.261). The differences of the interspace angle between L_2 _and L_3_, L_3 _and L_4 _before and after surgery were not significant(t = 1.309, P = 0.321; t = 0.299, P = 0.768), while it's significant for the angle between L_4 _and L_5_(t = 3.517, P = 0.017). During follow-up, the interspace angle became larger than that after surgery (Fig. 2), and the difference was significant (t = 2.684, P = 0.012). The coronal Cobb angle of the main curves were also larger than those after surgery, and the differences were also significant (t = 5.58, P = 0.000). The interspace angle after surgery was correlated with that on Bending films (r = 0.418, P = 0.022), and the interspace angle at final follow-up was correlated with that after surgery (r = 0.625, P = 0.000). There was moderate correlation between the loss of the interspace angle and the loss of coronal Cobb angle of the main curve during follow-up(r = 0.483, P = 0.007)(Table 3). There was no significant difference of the interspace angle after surgery, at final follow-up or the loss of interspace angle between the patients with single curve or double curves (2.6° vs. 1.4°, t = 0.452, P = 0.654; 3.2° vs. 5.4°, t = -0.665, P = 0.511; 3.6° vs. 3.2°, t = 0.177, P = 0.860). The interspace angle after surgery, at final follow-up and the loss of interspace angle were all larger in patients with LIV located at one vertebra above the lower end vertebra than those with LIV located at lower end vertebra, and the differences were all significant (Table 4).

**Table 2:** Changes of the interspace angle before and after surgery, and at final follow-up(X ± S)

location	cases	pre-operation	on Bending film	post-operative	follow-up
L2,3	3	1.7° ± 2.9°	-1.0° ± 3.5°	5.7° ± 4.0°	6.3° ± 2.9°
L3,4	21	2.8° ± 3.1°	-1.9° ± 6.7°	2.4° ± 6.1°	6.7° ± 8.1°
L4,5	6	5.5° ± 2.5°	-4.5° ± 4.6°	-2.3° ± 5.8°	-2.2° ± 5.2°
total	30	3.2° ± 3.1°	-2.3° ± 6.1°	1.8° ± 6.2°	4.9° ± 7.9°

**Table 3:** The changes and correlations of the coronal Cobb angle and the interspace angle before and after surgery, and at final follow-up (X ± S)

	preoperative	postoperative	at final follow-up	Loss
Coronal Cobb angle	48.9° ± 9.2°	11.7° ± 8.8°	18.1° ± 11.2°	6.3° ± 6.2°
Interspace angle	3.2° ± 3.1°	1.8° ± 6.2°	4.9° ± 7.9°	2.4° ± 4.7°
Correlation(r)	-0.067	-0.060	0.242	0.485
P	0.727	0.751	0.198	0.007

**Table 4:** Comparision of changes of the interspace angle between patients with LIV located at one vertebra above the lower end vertebra(group 1) and those with LIV located at lower end vertebra(group 2) (X ± S)

interspace angle	Group 1(n = 10)	Group 2(n = 20)	t value	P value
preoperative	3.1° ± 3.0°	3.3° ± 3.3°	0.161	0.873
postoperative	4.5° ± 3.8°	0.4° ± 6.7°	2.129	0.042
at final follow-up	9.3° ± 6.4°	2.6° ± 7.9°	2.316	0.028
Loss	4.8° ± 3.3°	1.2° ± 4.9°	2.073	0.047

## Discussion

In AIS patients with a thoracolumbar or lumbar curve, anterior correction and fusion surgery has several advantages compared with that of posterior approach: 1) the corrective force is applied at the greatest distance from the center of the curve in both lateral displacement and rotation, thus can provide stronger correction power[1,8,9]; 2) the spine is shortened, as opposed to lengthening in posterior approach, thus the risk of traction injury to the spinal cord is reduced[1]; 3) saving segments, more mobile segments can be preserved and the preservation of additional motion segments can reduce the risk of degenerative changes caudal to the fusion thus would be potentially more effective in decreasing the incidence of low back pain [10-13]; 4) preventing crankshaft phenomenon in immature children[1]. In addition, with the development of instrument technique overcoming the disadvantages of the implant, the surgeons becoming more familiar with the anterior approach, and thus the results of using anterior approach to treat AIS patients with a thoracolumbar or a lumbar curve become much better.

After anterior surgery, the lowest instrumented vertebra(LIV) usually cannot be paralleled to the caudal vertebra, so there will be an angle between the inferior endplate of the LIV and the superior endplate of the caudal vertebra, which is called interspace angle, and some doctors called it disc wedging or disc angulation[1,2,14,15]. Majd et al [1] reported that they treated 22 AIS patients with anterior surgery, with a coronal Cobb angle of 45° to 90°, the mean preoperative interspace angle was 10°, and after surgery, it was corrected to 2°, which was significantly corrected(P = 0.0001). They thought the larger the interspace angle, the more shear stress on the next caudal vertebra and increased risk of degeneration in future. The correction of the interspace angle could reduce the occurrence of future low back pain and degeneration. Satake et al[2] reported 61 patients of thoracolumbar or lumbar adolescent idiopathic scoliosis, the preoperative disc angle was 4.49° ± 5.48°, and it turned to -5.85° ± 4.37° post-operatively. They concluded that the preoperative disc angle and preoperative side-bending film disc angle had significant linear correlation with the postoperative disc angle. They also suggested that the compressive force applied to secure the intervertebral implants tend to create wedging below the LIV by pulling the LIV closer to the apex. Kaneda et al[14] reported postoperative disc wedging in patients with thoraolumbar or lumbar AIS following anterior fusion with instrumentation using Kaneda dual rod. In their study, the mean disc wedging angle was 6.6° in patients who underwent a short fusion and 3.0° in patients with lower end vertebra fused. They suggested that a more cephalic LIV and a shorter fusion created a larger disc wedging below the LIV. In our study, the postoperative discs angle was not correlated with the preoperative disc angle(r = -0.025, P = 0.894), but correlated with preoperative side-bending film disc angle (r = 0.418, P = 0.022). The disc angle at final follow-up was correlated with that after operation(r = 0.625, P = 0.000). In our study, however, the differences of the disc angle after operation between LIV at L_2_, L_3_, L_4 _were not significant.

The disc wedging can also occur in patients underwent posterior approach. Stasikelis et al[15] reported 29 cases of King type II and 11 cases of King type IV that underwent posterior or combined anterior and posterior correction with at least 1 year follow-up. In the 15 King type II patients and the 5 King type IV patients with posterior approach, the correction rates were 47.3% and 69.0%, and the interspace angle at final follow-up were 4.3° ± 4.4° and 3.4° ± 3.4°. While in the 14 King type II patients and the 6 King type IV patients with combined anterior and posterior approach, the correction rates were 77.7% and 95.5%, and the interspace angle at final follow-up were 8.7° ± 5.0° and 7.5° ± 8.3°. The interspace angle after surgery was 8.4° ± 6.0° in the patients with combined anterior and posterior approach, while it was 4.1° ± 4.1° in the patients with posterior approach, and the difference was significant (P < 0.01). From their study, we can see that the correction rate in the patients with combined anterior and posterior approach was better than that of the patients with posterior approach, but the interspace angle after surgery and at final follow-up were also larger, which was the reason they suggested that overcorrection of the upper lumbar curve was the cause of the increased interspace angle.

In the current study, the mean correction rate was76.1%, the interspace angle after surgery was improved, but the difference was not significant. At final follow-up, the interspace angle became larger than that of after surgery, while the coronal Cobb angle of the main curve was also became larger. There was moderate correlation between loss of interspace angle and coronal Cobb angle(r = 0.483) and no significant difference of the interspace angle between the patients with single curve or double curves. On convex side-bending films, the direction of the interspace angle can turn to the other side, so the flexibility of this segment is very ideal. However, as the interspace angle after surgery was larger than that on convex side-bending film, the discs could not change according to the position of the LIV. During follow-up, the interspace angle became even larger. We would suggest that it was due to the fact that the simultaneously correction of the upper curve in patients with double curves and the coronal balance were achieved at the loss of the interspace angle. In this study, 3 rib struts are used for bone graft and there were only 2 cases with loss of the fusion block after surgery, as we showed in figure 2. Therefore, it can be concluded that the loss of the coronal Cobb angle was mainly due to the loss of the interspace angle and this may be due to the shorter fusion range, while not due to pseudoarthrosis. Although the loss of the coronal Cobb angle and the interspace angle were a little significant, the fusion level would not extended as the global coronal balance was all satisfactory. Satake et al also noted this point. Although this change is known to be caused by overcorrection of the upper lumbar curve, it is still unknown whether there are other reasons and how to prevent this phenomenon. However, we should not plan to reduce the interspace angle through decreasing the correction rate of the curve. For posterior approach, pedicle screws are used at the LIV, thus distraction in the concave side and compression in the convex side can be performed and the two pedicles of the LIV are more leveled, and this may reduce the interspace angle.

## Conclusion

The interspace angle could be improved after anterior correction and instrumentation surgery, but it became larger during follow-up. The loss of the interspace angle was correlated with the loss of coronal Cobb angle of the main curve during follow-up.

Till now, the changes of the interspace angle after anterior correction and instrumentation are only a radiographic finding. It's a new phenomenon which is just observed, and without experimental data and long time follow-up with large sample. The definite reason, natural history and the significance of the disc wedging is unknown. From our study, the patients don't have any symptom and it's just a radiographic appearance. But the follow-up time is a little shorter and further investigation is needed.

## Abbreviations

AIS: adolescent idiopathic scoliosis

AP: anteroposterior

AVR: apical vertebral rotation

AVT: apical vertebral translation

CB: coronal balance

CDH: Cotrel-Dubousset Horizon

CSVL: central sacral vertical line

LIV: lowest instrumented vertebra

TSRH: Texas Scottish Rite Hospital instrument

## Supplementary Material

Additional file 1 Chinese version of the manuscript. The file is the Chinese version of the revised manuscript. Click here for file
