# Supplementary material for: The changes of the interspace angle after anterior correction and instrumentation in adolescent idiopathic scoliosis patients
Source: J Orthop Surg Res. 2007 Oct 29;2:17. doi: 10.1186/1749-799X-2-17 (PMC2198907; doi:10.1186/1749-799X-2-17)
Supplement: Additional file 1 — Chinese version of the manuscript. The file is the Chinese version of the revised manuscript. [file 1749-799X-2-17-S1.doc]

青少年特发性脊柱侧凸前路矫形、固定融合术后椎间角的变化

王以朋，邱贵兴， 于斌 ，仉建国，李佳忆，翁习生，沈建雄，费琦，李其一，

中国医学科学院 中国协和医科大学 北京协和医院骨科 北京 100730

**[摘要]**

目的 探讨青少年特发性脊柱侧凸前路矫形、固定融合术后椎间角的变化。

**方法** 回顾性分析接受前路矫形、固定融合术的青少年特发性脊柱侧凸患者的临床及影像学资料，测量、分析手术前后侧凸的Cobb角、矫形率、冠状面平衡、下融合椎旋转度、椎间角的变化。

**结果** 共30例患者，手术前、后主弯（胸腰弯/腰弯）冠状面Cobb角平均分别为48.9° 和11.7°，矫形率平均为76.1%。术前下融合椎旋转度平均为2.1度，术后改善到1.2度。术前、Bending像、术后、随访时椎间角平均分别为3.2°、-2.3°、1.8°和4.9°。手术前后椎间角比较差异无统计学意义（P=0.261），随访时椎间角较术后增大，且差异有统计学意义（P＝0.012）。术后椎间角同Bending像椎间角相关（r＝0.418，P=0.022），随访时椎间角同术后椎间角相关（r＝0.625，P=0.000）。随访时椎间角的增大同主胸弯矫形的丢失相关（r=0.483，P=0.007）。

**结论** 前路矫形、固定融合术可使椎间角得到改善，但在随访时此角度又有丢失，随访时椎间角的增大同主胸弯矫形的丢失有关。

**关键词** 特发性脊柱侧凸；前路融合；椎间角；下融合椎；

**引言**

随着人们对特发性脊柱侧凸认识的增加，其治疗越来越规范化。不同类型的脊柱侧凸其手术入路也不一样，胸腰段和腰段脊柱侧凸多选择前路手术。前路矫形内固定术后，下融合椎（lowest instrumented vertebra LIV）下终板与下方相邻椎体上终板之间椎间隙的开角称为椎间角（interspace angle）[1,2]，有文献报导此角常较术前增大，但无详细的文献分析。本文总结我院1998年11月～2003年5月收治的特发性脊柱侧凸接受前路矫形内固定的患者，分析椎间角的变化情况。

**材料和方法**

回顾性分析我科自1998年11月～2003年5月行前路矫形内固定的特发性脊柱侧凸病例，入选标准如下：（1）侧凸为特发性，椎体无先天性畸形；（2）青少年患者，年龄不超过18岁；（3）侧凸类型为胸腰弯或腰弯(PUMC Ib/Ic型；Lenke 5型)；或者胸腰双弯但胸弯较柔韧，适合单纯行前路手术仅固定腰弯(PUMC IIc1/IId1型；Lenke 5型) [3,4]；（4）单一前路矫形固定融合；（5）至少随访半年以上。

通过对术前站立正侧位相、仰卧位左右Bending相、术后及随访时站立正侧位相的测量，记录冠状面及矢状面Cobb角、侧凸柔韧性、侧凸矫形率、顶椎旋转度、顶椎偏距、冠状面平衡、下融合椎旋转度、术前椎间角、Bending像上椎间角及术后和随访时的椎间角变化。如果椎间角开角方向同侧凸主弯凸侧方向一致定义此椎间角为正值，负责为负值。顶椎旋转度按Nash-Moe方法测量[5]，0度：双侧椎弓根对称，无旋转；I度：凸侧椎弓根移向中线，但未超出第一格，凹侧椎弓根变小；II度：凸侧椎弓根进一步向内侧已移至第二格，凹侧椎弓根消失；III度：凸侧椎弓根移至中央，凹侧椎弓根消失；IV度：凸侧椎弓根越过中央，靠近凹侧。根据SRS定义，顶椎偏距是指在站立位正侧位相上，侧凸顶椎中点到骶正中线的垂直距离（mm），冠状面平衡是指骶骨中点至经C7铅垂线的垂直距离（mm）[6]。

本组资料所使用的统计软件为SPSS 10.0 for Windows，统计方法为t检验，P值<0.05时认为有统计学差异。

**结果**

共有30例患者符合入选标准，其中男性4例，女性26例，平均年龄14.8岁(10~18岁)，平均随访17.7月（6~24月）。脊柱侧凸中单弯8例，双弯22例，侧弯类型PUMC Ib 3例，Ic 5例，IIc1 2例， IId1 20例（图1）。主侧凸向左25例，向右5例。手术选用胸腹联合切口或腹膜后切口行前路侧凸矫形内固定植骨融合术，术中均行标准的去旋转操作。融合节段范围选择遵循Hall原则，要求融合区上下椎间盘活动性良好 [7]。手术所使用的矫形器械包括TSRH 9例，CDH 12例，MosMiami 8例，Isola 1例。融合节段：T10-L2 2例，T11-L2 1例，T10-L3 2例，，T11-L3 4例， T12-L3 15例，T12-L4 6例。下融合椎位于L2 3例，L3 21例，L4 6例。椎间角位置：L2-3 3例，L3-4 21例，L4-5 6例。

术前主弯冠状面Cobb角平均48.9°（33°～62°），反向Bending像冠状面Cobb角平均16.1°（-15°～40°），侧弯柔韧性平均67.2％（31.0％～100％）。双弯者次弯冠状面Cobb角平均31.3°（20°～48°），反向Bending相冠状面Cobb角平均12.1°（2°～24°），侧弯柔韧性平均62.0％（16.7％～92.9％）。术后主弯冠状面Cobb角平均11.7°（-1°～36°），矫正率平均76.1％（40％～100％）。双弯者术后次弯冠状面Cobb角平均19.1°（10°～32°），自动矫正率平均38.8％（3.0％～60％）。术后主弯冠状面Cobb角、AVR和AVT均得到明显改善，具体见表1。

表1：手术前后主弯各参数变化(Χ±S)

|  | 术前 | 术后 | t 值 | P 值 |
| --- | --- | --- | --- | --- |
| 冠状面Cobb角 | 48.9°±9.2° | 11.7°±8.2° | 19.76 | 0.000 |
| 冠状面平衡(mm) | 14.1±12.0 | 15.6±13.0 | 0.66 | 0.516 |
| AVR(degree) | 2.1±0.6 | 1.2±0.5 | 9.36 | 0.000 |
| AVT(mm) | 43.3±13.2 | 13.4±9.0 | 10.63 | 0.000 |

手术前后椎间角的变化见表2。 术前椎间角平均3.2°，术后改善至1.8°，但此差异无统计学意义（t=1.146，P＝0.261）。其中L2-3椎间角、L3-4椎间角术后比术前改善不明显（t=1.309， P=0.321； t=0.299， P=0.768）；L4-5椎间角术后比术前明显改善（t=3.517，P＝0.017）。在随访时，椎间角比术后增大（图2），差异也具有统计学意义（t=2.684，P＝0.012）。随访时主弯冠状面Cobb角也比术后加大，差异也具有统计学意义（t=5.58，P=0.000）。术后椎间角同Bending像上椎间角相关（r＝0.418, P=0.022），随访时椎间角同术后椎间角相关（r＝0.625, P=0.000）。随访时椎间角的丢失同主弯冠状面的丢失存在相关性（r=0.483, P=0.007）（表3）。单弯与双弯患者的椎间角在术后及随访时以及椎间角的丢失方面的差异均无统计学意义（2.6° vs. 1.4°, t=0.452, P=0.654; 3.2° vs. 5.4°, t=-0.665, P=0.511; 3.6° vs. 4.0°, t=0.177, P=0.860）。下融合椎在下端椎上一椎体与下融合椎在下端椎的患者相比，其椎间角在术后及随访时以及椎间角的丢失方面均更大，差异均有统计学意义（表4）。

表2：手术前、后及随访时椎间角的变化 (±S)

| 位置 | 病例数 | 术前 | Bending 像 | 术后 | 随访时 |
| --- | --- | --- | --- | --- | --- |
| L2,3 | 3 | 1.7°±2.9° | -1.0°±3.5° | 5.7°±4.0° | 6.3°±2.9° |
| L3,4 | 21 | 2.8°±3.1° | -1.9°±6.7° | 2.4°±6.1° | 6.7°±8.10° |
| L4,5 | 6 | 5.5°±2.5° | -4.5°±4.6° | -2.3°±5.8° | -2.2°±5.2° |
| total | 30 | 3.2°±3.1° | -2.3°±6.1° | 1.8°±6.2° | 4.9°±7.9° |

表3：手术前、后及随访时冠状面Cobb角和椎间角的变化及相关性 (±S)

|  | 术前 | 术后 | 随访 | 丢失 |
| --- | --- | --- | --- | --- |
| 冠状面Cobb角 | 48.9°±9.2° | 11.7°±8.8° | 18.1°±11.2° | 6.3°±6.2° |
| 椎间角 | 3.2°±3.1° | 1.8°±6.2° | 4.9°±7.9° | 2.4°±4.7° |
| 相关性(r) | -0.067 | -0.060 | 0.242 | 0.485 |
| P | 0.727 | 0.751 | 0.198 | 0.007 |

表4：下融合椎在下端椎上一椎体（组1）与下融合椎在下端椎（组2）的患者椎间角的变化比较(±S)

| 椎间角 | 组 1(n=9) | 组2(n=21) | t值 | P 值 |
| --- | --- | --- | --- | --- |
| 术前 | 3.1°±3.0° | 3.3°±3.3° | 0.161 | 0.873 |
| 术后 | 4.5°±3.8° | 0.4°±6.7° | 2.129 | 0.042 |
| 随访时 | 9.3°±6.4° | 2.6°±7.9° | 2.316 | 0.028 |
| 椎间角丢失 | 4.8°±3.3° | 1.2°±4.9° | 2.073 | 0.047 |

**讨论**

对胸腰段或腰段AIS患者施行前路手术相对于后路手术有许多优势：（1）矫形力量优于后路[1,8,9]；（2）脊柱缩短，脊髓损伤危险降低[1]；（3）减少融合节段，保留更多的运动节段，从而降低融合区远端脊柱的退变及腰背痛的发生[10~13]；（4）骨骼发育未成熟患儿远期发生曲轴现象危险降低[1]。随着内固定器械的发展克服早期前路手术器械的缺陷，加上脊柱外科医生对前路手术技术也逐渐熟悉，对合适病例施行前路矫形固定融合术的效果越来越好。

脊柱侧凸前路矫形手术后，由于下方融合椎在术后很难完全平行于相邻的下方椎体，因此在下方融合椎（lowest instrumented vertebra）的下终板与相邻的下方椎体的上终板之间存在一定的角度，称之为椎间角（interspace angle），也有人称之为椎间盘楔形变（disc wedging）或椎间盘成角（disc angulation）[1,2,14,15]。Majd[1]等报告22例AIS患者接受前路手术治疗，冠状面Cobb角45°～90°，术前椎间角平均10°，术后改善至2°，较术前明显改善(P=0.0001)。他们认为术后此角度越大，对下方椎体的剪切应力越大，远期退变的危险也增加。同时他们认为此角度的改善可减少远期腰背痛及退变的发生。Satake等[2] 报告61 例接受前路手术治疗的特发性胸腰弯/腰弯患者，术前椎间角为4.49°±5.48°，术后为-5.85°±4.37° 。他们总结认为术后椎间角同术前椎间角及Bending像上椎间角存在相关性。同时他们认为矫形操作时的加压力会在下融合椎下方产生楔形变，把下融合椎更向顶椎牵拉。Kaneda等[14] 报告使用Kaneda双棒对特发性胸腰弯/腰弯患者施行前路矫形手术，在他们的报告中，接受短节段融合的患者术后椎间角平均为6.6°，而将下端椎包括在融合范围内的患者术后椎间角平均为3.0°。他们认为下融合椎越靠近头侧，融合节段越短，在下融合椎下产生的椎间角越大。在本组研究中，术后椎间角同术前椎间角无明显相关性（r＝－0.025, P=0.894），但同Bending像上椎间角存在相关性（r＝0.418, P=0.022）。随访时的椎间角同术后椎间角存在相关性（r＝0.625, P=0.000）。但下融合椎在L2 、L3和L4的不同患者相比，术后椎间角差异无统计学意义。

在后路矫形手术后，同样也存在椎间盘成角问题。Stasikelis等[15]报告29例King II，11例King IV AIS患者接受后路手术或前后路联合手术矫形内固定治疗，术时年龄10～20岁，最短随访时间1年。后路手术治疗的15例King II和5例King IV患者患者冠状面矫形率分别为47.3%和69.0%，随访时椎间角分别为4.3°±4.4°和3.4°±3.4°。而在前后路联合手术治疗的14例King II患者和6例King IV患者的冠状面矫形率分别为77.7%和95.5%，随访时椎间角分别为8.7°±5.0°和7.5°±8.3°。前后路联合方法术后椎间角为8.4°±6.0°，仅行后路者为4.1°±4.1°，二者之间存在显著差异（P<0.01）。从中可以看出，施行前后路手术组比单纯行后路手术组矫形率要大，但同时术后及随访时其椎间角也大，因此他们认为上腰椎侧凸的过度矫正是导致下腰椎椎间角增加的原因。

本组资料显示，前路手术矫形率平均为76.1%，术后椎间角得到改善，在随访时，椎间角比术后增大，主弯冠状面Cobb也增大，且在二者间存在相关性 （r=0.483）。在随访时，单弯与双弯患者比较，椎间角及椎间角丢失的差异均无统计学意义。在术前Bending相上，椎间角的朝向大部分患者可反向张开，说明此节段间盘柔韧性良好，但术后椎间角的度数均较Bending相上的角度大，说明椎体并不能如我们想象的会随着下融合椎的位移而产生相应的变化。在随访时，此角度比术后又进一步增大，考虑原因是由于双弯患者上弯的自动矫正以及患者的躯干平衡是以牺牲下方椎间角为代价完成的。我们使用3段肋骨条植骨，术后仅2例患者发生融合固定节段角度的丢失（图2）。因此，我们认为冠状面Cobb角的丢失主要同椎间角的丢失有关，这可能是由于融合节段较短所致，而不是源于假关节形成。虽然，冠状面Cobb角和椎间角的丢失有些明显，但我们仍不想延长融合节段，因为患者的整体冠状面平衡很理想。Satake等[2]也注意到了这一点。虽然现在知道这一变化是由于上腰椎侧凸的过度矫正导致的，但是否还有其它原因及如何预防这一现象，尚无相关文献报道，目前还不能通过减小侧凸的矫形率这一方法来降低椎间角。另外，后路手术在下方融合椎使用双侧椎弓根螺钉，从而可以在凹侧撑开，凸侧加压，以使双侧螺钉尽量保持水平，这样也可以使椎间角减小。

目前，前路矫形融合术后椎间角的变化还仅是一种影像学表现，国际上也是刚刚注意到这个问题，并没有实验室的相关数据，也没有大样本的长期随访。这一现象的自然病史还不清楚。从本组资料来看，所有椎间角增大的患者均无临床不适，仅为一种影像学表现，但由于随访时间尚短，其临床意义尚待进一步随诊观察。

参考文献：

1. Majd ME, Castro FP Jr, Holt RT. Anterior fusion for idiopathic scoliosis. Spine, 2000, 25: 696-702.
2. [Satake K](http://www.ncbi.nlm.nih.gov/sites/entrez?Db=pubmed&Cmd=Search&Term="Satake K"%5BAuthor%5D&itool=EntrezSystem2.PEntrez.Pubmed.Pubmed_ResultsPanel.Pubmed_RVAbstractPlus), [Lenke LG](http://www.ncbi.nlm.nih.gov/sites/entrez?Db=pubmed&Cmd=Search&Term="Lenke LG"%5BAuthor%5D&itool=EntrezSystem2.PEntrez.Pubmed.Pubmed_ResultsPanel.Pubmed_RVAbstractPlus), [Kim YJ](http://www.ncbi.nlm.nih.gov/sites/entrez?Db=pubmed&Cmd=Search&Term="Kim YJ"%5BAuthor%5D&itool=EntrezSystem2.PEntrez.Pubmed.Pubmed_ResultsPanel.Pubmed_RVAbstractPlus), [Bridwell KH](http://www.ncbi.nlm.nih.gov/sites/entrez?Db=pubmed&Cmd=Search&Term="Bridwell KH"%5BAuthor%5D&itool=EntrezSystem2.PEntrez.Pubmed.Pubmed_ResultsPanel.Pubmed_RVAbstractPlus), [Blanke KM](http://www.ncbi.nlm.nih.gov/sites/entrez?Db=pubmed&Cmd=Search&Term="Blanke KM"%5BAuthor%5D&itool=EntrezSystem2.PEntrez.Pubmed.Pubmed_ResultsPanel.Pubmed_RVAbstractPlus), [Sides B](http://www.ncbi.nlm.nih.gov/sites/entrez?Db=pubmed&Cmd=Search&Term="Sides B"%5BAuthor%5D&itool=EntrezSystem2.PEntrez.Pubmed.Pubmed_ResultsPanel.Pubmed_RVAbstractPlus), [Steger-May K](http://www.ncbi.nlm.nih.gov/sites/entrez?Db=pubmed&Cmd=Search&Term="Steger-May K"%5BAuthor%5D&itool=EntrezSystem2.PEntrez.Pubmed.Pubmed_ResultsPanel.Pubmed_RVAbstractPlus). Analysis of the lowest instrumented vertebra following anterior spinal fusion of thoracolumbar/lumbar adolescent idiopathic scoliosis: can we predict postoperative disc wedging? Spine, 2005; 30:418-426.
3. [Qiu G, Zhang J, Wang Y, Xu H, Zhang J, Weng X, Lin J, Zhao Y, Shen J, Yang X, Luk KD, Lu D, Lu WW.](http://www.ncbi.nlm.nih.gov/sites/entrez?Db=pubmed&Cmd=ShowDetailView&TermToSearch=15959372&ordinalpos=45&itool=EntrezSystem2.PEntrez.Pubmed.Pubmed_ResultsPanel.Pubmed_RVDocSum)A new operative classification of idiopathic scoliosis: a Peking union medical college method. Spine, 2005;30:1419-1426.
4. [Lenke LG, Betz RR, Harms J, Bridwell KH, Clements DH, Lowe TG, Blanke K.](http://www.ncbi.nlm.nih.gov/sites/entrez?Db=pubmed&Cmd=ShowDetailView&TermToSearch=11507125&ordinalpos=11&itool=EntrezSystem2.PEntrez.Pubmed.Pubmed_ResultsPanel.Pubmed_RVDocSum)Adolescent idiopathic scoliosis: a new classification to determine extent of spinal arthrodesis. J Bone Joint Surg(Am), 2001:83:1169-1181.
5. Nash CL Jr, Moe JH. A study of vertebral rotation. J Bone Joint Surg(AM), 1969, 51:223-229.
6. The Working Group on 3-D Classification (Chair Larry Lenke, MD), and the Terminology Committee. SRS Terminology Committee and Working Group on Spinal Classification:Revised Glossary of Terms. Available at: http://www.srs.org/professional/glossary/glossary.asp.Accessed Jun 1, 2007
7. Hall JE, Millis MB, Snyder BD. Short segment anterior instrumentation for

thoracolumbar scoliosis. In: Bridwell KH, DeWald RL, eds. The Textbook of Surgery. 2nd ed. Philadelphia, PA: Lippincott Williams & Wilkins; 1997:665–74.

1. Dwyer AF, Newton NC, Sherwood AA. An anterior approach to scoliosis: a preliminary report. Clin Orthop, 1969; 62: 192-202.
2. [Luk KD, Leong JC, Reyes L, Hsu LC.](http://www.ncbi.nlm.nih.gov/sites/entrez?Db=pubmed&Cmd=ShowDetailView&TermToSearch=2711242&ordinalpos=26&itool=EntrezSystem2.PEntrez.Pubmed.Pubmed_ResultsPanel.Pubmed_RVDocSum)The comparative results of treatment of idiopathic thoracolumbar and lumbar scoliosis using Harrington, Dwyer, and Zielke instrumentation. Spine, 1989; 14:275-280.
3. Ginsburg HH, Goldstein L, Haake PW, Perkins S, Gilbert K. Longitudinal study of back pain in postoperative idiopathic scoliosis: Long-term follow-up: Phase IV. Presented at Scoliosis Research Society 30th Annual Meeting, September 13–16, 1995, Asheville, North Carolina: Paper 48.
4. Bradford DS. Anterior spinal surgery in the management of scoliosis: Indications-techniques-results. Orthop Clin North Am 1979;10:801-12.
5. Gaines RW, Leatherman KD. Benefits of the Harrington compression system in lumbar and thoracolumbar idiopathic scoliosis in adolescent and adults. Spine 1981;6:483-8.
6. Cochran T, Irstan L, Nachemson A. Long term anatomic and functional changes in patients with adolescent idiopathic scoliosis treated by Harrington rod fusion. Spine 1983;8:576-84.
7. Kaneda K. Shono Y. Satoh S. Abumi K. New anterior instrumentation for the management of thoracolumbar and lumbar scoliosis: application of the Kaneda two-rod system. Spine, 1996, 21:1250-1261.
8. Stasikelis PJ. Miller WD. Wilson C. Pugh LI. Allen BL Jr. Spine behavior caudal to instrumentation in King II and IV curves.

Clin Orthop Relat Res, 2002, (400):132-139.

图例：

图1. 16岁女孩，青少年特发性脊柱侧凸，PUMC IId1型。术前X线片显示左腰弯39º，右胸弯20º，腰弯顶椎位于L_2,3_ 椎间盘，术前侧位X线片显示无胸腰段后凸（A, B）。L_4_ 和L_5_ 间的椎间角是4º，在右Bending像上，椎间角为9º (C)，而在左Bending像上改善到0º(D)。手术采用前路矫形固定融合，融合节段从T_12_ 到L4。术后X线片显示矫形良好，椎间角改善到0º (E, F)。 术后3年随访时，椎间角增加到2º，冠状面平衡良好(G, H)。

图2. 17岁女孩，青少年特发性脊柱侧凸，PUMC Ic型。术前X线片显示左腰弯52º，顶椎位于L_1,2_ 椎间盘（A, B）。L_3_ 和L_4_ 间的椎间角是-5º，在右Bending像上，椎间角为0º (C)，而在左Bending像上仍为-5º(D)。Bending像上T_12_-L_1_和L_2,3_ 椎间盘未张开，因此前路矫形固定融合节段从T_12_ 到 L_3_。术后X线片显示矫形良好，椎间角改善到0º (E, F)。术后9月后随访时，矫形有丢失，椎间角增大到7º (G, H)。
